# Investigation and identification of protein γ-glutamyl carboxylation sites

**DOI:** 10.1186/1471-2105-12-S13-S10

**Published:** 2011-11-30

**Authors:** Tzong-Yi Lee, Cheng-Tsung Lu, Shu-An Chen, Neil Arvin Bretaña, Tzu-Hsiu Cheng, Min-Gang Su, Kai-Yao Huang

**Affiliations:** 1 Department of Computer Science and Engineering, Yuan Ze University, Chung-Li 320, Taiwan

## Abstract

**Background:**

Carboxylation is a modification of glutamate (Glu) residues which occurs post-translation that is catalyzed by γ-glutamyl carboxylase in the lumen of the endoplasmic reticulum. Vitamin K is a critical co-factor in the post-translational conversion of Glu residues to γ-carboxyglutamate (Gla) residues. It has been shown that the process of carboxylation is involved in the blood clotting cascade, bone growth, and extraosseous calcification. However, studies in this field have been limited by the difficulty of experimentally studying substrate site specificity in γ-glutamyl carboxylation. *In silico* investigations have the potential for characterizing carboxylated sites before experiments are carried out.

**Results:**

Because of the importance of γ-glutamyl carboxylation in biological mechanisms, this study investigates the substrate site specificity in carboxylation sites. It considers not only the composition of amino acids that surround carboxylation sites, but also the structural characteristics of these sites, including secondary structure and solvent-accessible surface area (ASA). The explored features are used to establish a predictive model for differentiating between carboxylation sites and non-carboxylation sites. A support vector machine (SVM) is employed to establish a predictive model with various features. A five-fold cross-validation evaluation reveals that the SVM model, trained with the combined features of positional weighted matrix (PWM), amino acid composition (AAC), and ASA, yields the highest accuracy (0.892). Furthermore, an independent testing set is constructed to evaluate whether the predictive model is over-fitted to the training set.

**Conclusions:**

Independent testing data that did not undergo the cross-validation process shows that the proposed model can differentiate between carboxylation sites and non-carboxylation sites. This investigation is the first to study carboxylation sites and to develop a system for identifying them. The proposed method is a practical means of preliminary analysis and greatly diminishes the total number of potential carboxylation sites requiring further experimental confirmation.

## Introduction

Carboxylation is a post-translational modification (PTM) of glutamate (Glu) residues in proteins that is primarily involved in the blood clotting cascade specifically occurring in factors II, VII, IX, and X, protein C, protein S, as well in some bone proteins [1,2]. Vitamin K is a critical cofactor in the post-translational conversion of Glu residues to γ-carboxyglutamate (Gla) residues [3]. Carboxylation is catalyzed by γ-glutamyl carboxylase [4] and proceeds in the lumen of the endoplasmic reticulum [5]. The vitamin K-dependent carboxylase transforms Glu to Gla when carbon dioxide (CO_2_) is added at the γ-position in the presence of oxygen (O_2_) and reduced vitamin K [2]. The carboxylated proteins can be activated when Gla domain binds Ca^2+^[6]. Since Glu is a weak Ca^2+^ chelator and Gla is a much stronger one, the transformation by the vitamin K-dependent carboxylase greatly increases the Ca^2+^-binding capacity of a protein [7]. Studies conducted over the last few years have revealed that the γ-glutamyl carboxylated proteins in vertebrates can be categorized into three main groups [8]. The first group comprises the carboxylated proteins with an amino terminal Gla domain, and includes vitamin K-dependent blood coagulation factors and co-regulators of blood coagulation [9]. The second group is composed of osteocalcin and matrix Gla protein (MGP) [10,11], and includes three and five Gla residues, respectively, which are critical to the regulation of bone growth and extraosseous calcification [12,13]. The third group is the γ-glutamyl carboxylase, itself, which includes Gla residues [14].

Morris *et al.* used mass spectrometry to reveal the processive behavior of vitamin K-dependent carboxylation wherein multiple carboxylations occur during a single substrate binding event [6]. The efficient carboxylation of native substrates depends on the binding of a conserved region to the carboxylase with a submicromolar affinity constant [15,16]. Owing to the biological importance of γ-glutamyl carboxylation, more attention has been paid to mass spectrometric analyses in order to identify experimentally confirmed carboxylation sites. Nevertheless, *in vitro* identification of protein carboxylation sites requires a very large amount of time and effort. Meanwhile, *in silico* methods have the potential to characterize carboxylated sites before experiments are conducted. Additionally, *in silico* identification presents a more feasible means of preliminary analysis with the potential to significantly diminish the number of potential carboxylation sites requiring further experimental verification.

Given the importance of γ-glutamyl carboxylation in biological mechanisms, this work focuses on investigating the substrate site specificity of carboxylation sites. The experimentally validated γ-glutamyl carboxylations were mainly gathered from UniProtKB, a universal protein resource [17]. Numerous experimental carboxylation sites in humans have been taken from HPRD [18], which contains curated data that the authors manually extracted from the PubMed literature database. Amino acid side chains that undergo post-translational modification tend to be accessible on protein surfaces [19]. Therefore, this investigation not only examines the amino acid composition around carboxylation sites, but also considers structural characteristics, including solvent-accessible surface area (ASA) and secondary structure. In order to characterize the structural properties of the tertiary structures of carboxylated proteins, experimentally identified carboxylation sites are mapped to its corresponding positions from the protein sequences of Protein Data Bank (PDB) [20]. However, most of the collected carboxylation sites lack the corresponding PDB tertiary structures. Accordingly, two tools, RVP-Net [21,22] and PSIPRED [23], are utilized to calculate the ASA values and the secondary structures of amino acids in protein sequences, respectively.

Each feature is examined to evaluate its capacity to differentiate carboxylation sites from non-carboxylation sites. A support vector machine (SVM) is utilized to establish a predictive model with various features, including the positional weighted matrix of amino acids, amino acid composition, ASA, and secondary structure. Five-fold cross-validation is utilized to test the predictive performance of the generated SVM model. To prevent the generated model from an overestimation of predictive power, homologous sequences are eliminated by applying a window length of 2*n*+1 to carboxylation sites. After building and evaluating the model, the selected models providing the best accuracy are further tested using a data set of independent testing. The independent test set, which is not included in the training set, is then adopted to evaluate whether the selected model is over-fitting to the training data. Finally, the training features and window length providing the highest predictive accuracy for the model are used to construct a web-based system for identifying γ-glutamyl carboxylation.

## Material and methods

### Data collection and preprocessing

A comprehensive PTM resource dbPTM [24], which collects PTM data from release 15.0 of UniProtKB [17] and release 8.0 of HPRD [18], comprises of 1123 Gla residues in 182 protein entries from multiple organisms. After the non-experimental sites, annotated as “by similarity”, “potential” and “probable” in the “MOD_RES” fields of UniProtKB, have been removed, 463 experimental carboxylation sites from 134 carboxylated proteins are obtained. It is observed that the carboxylation site occurs on Glu residues. In this investigation, all carboxylated Glu residues are regarded as the positive set. On the other hand, Glu residues, found in the experimental carboxylated proteins, which are not annotated as carboxylation sites are regarded as the negative set. Consequently, a total of 854 non-carboxylated Glu sites are defined as negative set. This work aims to investigate the structural characteristics of protein carboxylation sites; with reference to a previous work [25], features such as amino acid composition, accessible surface area (ASA), and secondary structure are explored. The extracted features are used to construct a predictive model and are evaluated in terms of its ability to differentiate carboxylation sites from non-carboxylation sites.

To avoid an overestimation in the cross-validation, the removal of homologous sequences in the positive data is done by using a window size of 2*n*+1 centered on the carboxylation site. With reference to the method of homology reduction in N-Ace [26], two carboxylated proteins having more than 30% sequence similarity are regarded as homologous proteins. Then, for every two homologous protein sequences, BL2SEQ [27] is applied to re-align the fragment sequences with 2*n*+1 amino acids centered on the carboxylated sites. For two fragment sequences having a sequence similarity higher than 50%, only one fragment sequence is kept while the other is removed the positive set. The same approach is also applied to generate the non-homologous negative set.

In the evaluation of cross-validation, the constructed SVM models may be overestimated due to a possible over-fit to the training data set. Thus, about 15% of experimental carboxylated proteins are randomly chosen to construct an independent data set which is later used for estimating the actual prediction power of the selected model [28-30]. As shown in Table 1, the non-homologous training data comprises of 302 carboxylated glutamate residues (positive set of training data) and 567 non-carboxylated glutamate residues (negative set of training data) from 79 carboxylated proteins. Additionally, a total of 60 carboxylated glutamate residues and 99 non-carboxylated glutamate residues from 14 carboxylated proteins, respectively, are defined as the positive set and negative set for independent testing. After the *k*-fold cross-validation, the trained model yielding the best accuracy is evaluated using the independent test data.

**Table 1:** Statistics of experimentally verified carboxylation sites in training data and independent testing data.

Data set	**All data** (UniProt release 15.0 and HPRD 8.0)	**Training data** (non-homologous)	**Independent testing data** (non-homologous)
Number of carboxylated proteins	134	79	14
Number of carboxylated glutamate residues	463	302	60
Number of non-carboxylated glutamate residues	854	567	99

### Feature investigation

This work not only investigates the composition of amino acids that surround carboxylation sites, but also takes the solvent accessible surface area (ASA), and secondary structure (SS) into account. A window size of 2*n*+1 is utilized to extract fragment sequences from positive and negative sets of training data. Various values of *n* changing from four to ten are applied to decide the optimal window length. The amino acid composition (AAC) [31], which refers to the relative frequencies of twenty amino acids in a given window length, is regarded as the elementary feature in the investigation of carboxylation sites [28]. A vector of 20 elements for amino acid composition specifies the number of occurrences of the twenty amino acids normalized by the total number of residues in a fragmented sequence with window length 2*n*+1. To investigate the position-specific amino acid composition around the carboxylation sites, a positional weighted matrix (PWM) is determined using non-homologous positive set of training data [32]. The PWM specifies the occurring frequency of amino acids in each position of a fragment. A matrix of (2*n*+1)×*m* elements, where 2*n*+1 refers to the window length and *m* contains 21 elements that stands for the 20 amino acids and one terminal signal, is referred to in order to encode each fragment sequence in the training data.

The amino acids that undergoes post-translational modification were reported to be exposed on the surface of a protein [19]. Thus, the accessible surface area (ASA) surrounding the carboxylation sites is considered. Due to the fact that almost all of the experimental carboxylated proteins do not contain a corresponding PDB tertiary structure, RVP-Net [21,22] is utilized to calculate the ASA value, which is the percentage of the solvent-accessible area of each amino acid on a protein sequence. RVP-net is a prediction tool to determine the value of residual ASAs using neighborhood information and yields a mean absolute difference of 18.0 – 19.5% between the predicted and experimental values of ASA [22]. In this work, the full-length sequences of carboxylated proteins are submitted to RVP-Net to calculate the ASA value for all amino acids. The ASA values of the amino acids surrounding the carboxylation site are normalized to zero to one.

In investigating secondary structures surrounding carboxylation sites, PSIPRED [23] was utilized to predict the secondary structure from a given protein sequence. PSIPRED applied two feed-forward neural networks to predict secondary structure using the results from PSI-BLAST (Position Specific Iterated - BLAST) [33]. PSIPRED 2.0 has been reported as the top out of 20 evaluated methods by achieving a mean Q_3_ score of 80.6% for a test data containing 40 domains which have no significant similarity to PDB structures [34]. The full-length sequences of carboxylated proteins are submitted to PSIPRED to obtain the secondary structure of all amino acids. The resulted data of PSIPRED is encoded in terms of “H” for helix, “E” for sheet, and “C” for coil. In order to transform the three terms into numeric vectors, a three-dimensional binary vector is applied: helix (H) is encoded as “100,” sheet (E) is encoded as “010,” and coil (C) is encoded as “001”.

### Model learning and evaluation

In this study, support vector machine (SVM) is employed in order to create predictive models that utilize the explored features. In terms of binary classification, SVM adopts a kernel function to map samples into a higher dimensional space and subsequently determines a hyper-plane for effectively discriminating between the two classes of samples with a maximum margin and a minimum inaccuracy. LibSVM [35] is employed to generate a binary prediction model using the positive and negative training sets. The kernel function of SVM is radial basis function (RBF): . Moreover, two SVM parameters, cost and gamma value, are tuned to yield a highest accuracy.

*K*-fold cross-validation is an important method for evaluating the performance of a predictive model [36]. The training data is split into *k* approximately equal sized subgroups. For one round of *k*-fold cross-validation, *k*-1 subgroups are defined as the training set and the remaining one subgroup is defined as the validation set. The process of *k*-fold cross-validation is executed *k* times until each of the *k* subgroups regarded as the validation set one by one. In the evaluation of *k*-fold cross-validation, all data are equally considered as both the validation set and training set, and each data is used as the validation set exactly once [37]. In this study, *k* is set to five and the *k* results are integrated to produce a single estimation. The measurement of the predictive performance is defined as follows:(1)(2)(3)(4)(5)

where TP, FN, TN, and FP represent the numbers of true positives, false negatives, true negatives, and false positives, respectively. Since a two-class classification is of very different sizes, the Matthews correlation coefficient (MCC) is usually regarded as a balanced measure which takes into account the true and false positives and negatives [38]. The range of MCC values is between -1 and +1: a measure of +1 stands for a perfect prediction, 0 is a random prediction and -1 represents an inverse prediction. Furthermore, the parameters of a predictive model, such as window length, cost value of SVM, and gamma value of SVM, are optimized to achieve a highest accuracy. Finally, the SVM model yielding the highest accuracy is selected for further independent testing.

## Results and discussion

### Amino acid composition at the vicinity of carboxylation sites

**Figure 1:**
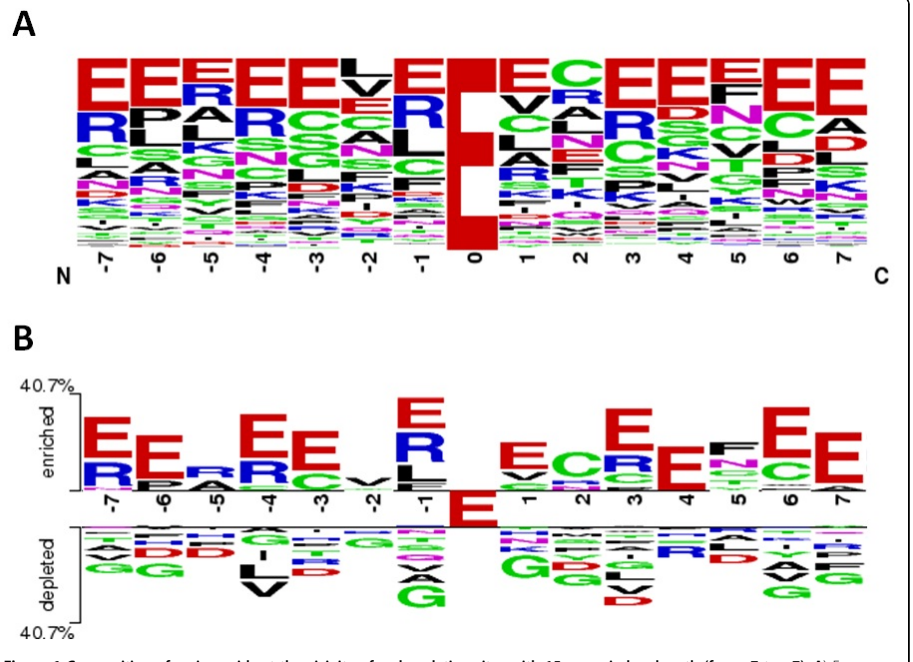
**Composition of amino acids at the vicinity of carboxylation sites with 15-mer window length (from -7 to +7). A**) Frequency plot of amino acid composition at each flanking position. **B**) Position-specific differences in amino acid composition at the vicinity of carboxylation sites (top) and non-carboxylation sites (bottom).

The aim of this work is to explore the substrate specificity of γ-glutamyl carboxylation sites. To examine the composition of amino acids around carboxylation sites, the amino acids from -7 to +7 that flank non-homologous carboxylation sites are graphically represented as sequence logos. WebLogo [39,40] is used to produce a frequency plot of sequence logo which shows the relative frequency of twenty amino acids at each position surrounding the carboxylation sites. This is applied to simplify the study of amino acid conservation near carboxylation sites. The frequency plot of the sequence logo in Fig. 1A presents the highly concentrated Glu residues around carboxylation sites. The high conservation of negatively charged glutamate residue is consistent with previous findings that the γ-carboxylation recognition site suffices to direct vitamin K-dependent carboxylation on an adjacent glutamate-rich region of thrombin in a propeptide-thrombin chimera [41].

To compare amino acid compositions between positive and negative data, the web-based tool, TwoSampleLogo [42], was utilized to detect significant differences in terms of the position-specific symbol compositions between two sets of multiple sequence alignments. Figure 1B shows the position-specific differences in amino acid composition between carboxylation sites (302 sequences) and non-carboxylation sites (567 sequences). With a 15-mer window length (from -7 to +7), the most prominent feature of carboxylation sites is the abundant and uniform Glu residue (negatively charged amino acid) that surrounds positions -7, -6, -4, -3, -1, +1, +3, +4, +6 and +7. Another interesting feature is the slight enrichment of arginine (positively charged amino acid) at positions -7, -5, -4, -1, and +3. The distant amino acids in the primary sequence, which may be immediately adjacent to the Gla residue in a three-dimensional structure, differ significantly between carboxylation and non-carboxylation sites. Also notable is the depleted Glu residue at flanking positions, particularly positions -2 and +2, which are close to the carboxylation sites.

### Structural characteristics at carboxylation sites

**Figure 2:**
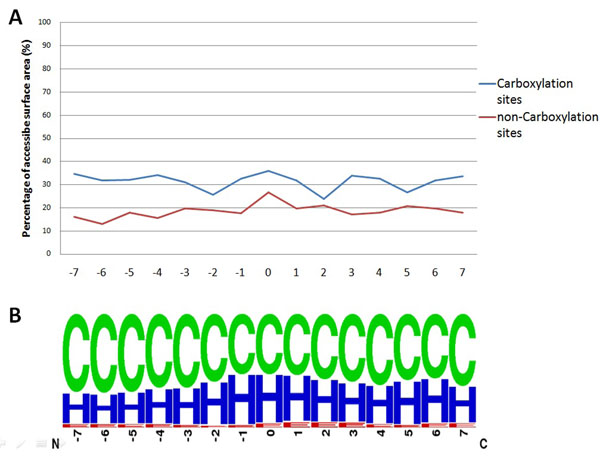
**Structural characteristics of carboxylation sites with 15-mer window length (from -7 to +7). A**) Comparison of average ASA percentage at carboxylation sites (blue line) and that at non-carboxylation sites (red line). **B**) Secondary structure around carboxylation sites.

As well as amino acid composition, the solvent-accessible surface area (ASA) and secondary structure (SS) of amino acids were considered to explore the structural characteristics of carboxylation sites. With regard to the application of RVP-Net [21,22], a previous investigation of protein methylation [25] demonstrated that the RVP-Net-computed ASA value is consistent with the observed values in PDB tertiary structures. Figure 2A presents the comparison of the mean ASA values between carboxylation sites (302 sequences) and non-carboxylation sites (567 sequences). The analysis reveals that the flanking region of carboxylation sites has a high preference for the solvent-accessible surface area, especially in the region from -1 to +1. The mean percentage of ASA on carboxylated glutamate residues is 37.8%, resulting in a great exposure to the solvent. In the investigation of ASA curves, the notable difference between carboxylation sites and non-carboxylation sites is found in the region from -7 to -4. Interestingly, the mean percentage of ASA is particularly low at positions -2 and +2, both of which are associated with Glu depletion. Figure 2B indicates that the flanking regions of carboxylated sites are probably present in the secondary structures of coil (loop) and helix. For carboxylated glutamate residues, 53% of the sites are located in coil structure, 42.6% are located in helical structure, and 4.4% are located in sheet structure.

### Determination of optimal window size based on amino acid composition

**Figure 3:**
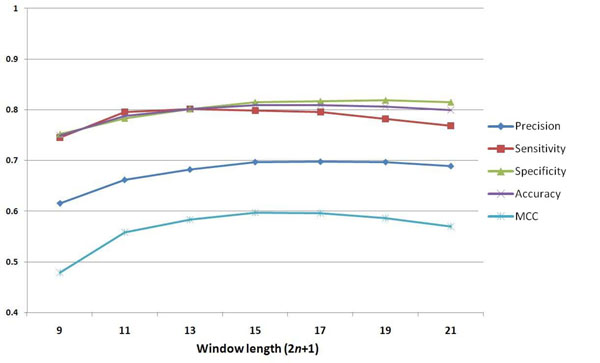
**Five-fold cross-validation performance of models trained using amino acid composition with varying window lengths.** To determine which window sizes can be used for generating the predictive model that best identifies carboxylation sites, a five-fold cross-validation is conducted to evaluate the models trained with different window lengths 2*n*+1, where *n* changes from four to ten.

To decide which window sizes can be used for generating the predictive model that best identifies carboxylation sites on Glu residues, a five-fold cross-validation is conducted to evaluate the models trained with different window lengths 2*n*+1, where *n* changes from four to ten. With reference to a previous work that determined the optimal window size for the identification of protein acetylation [26], the amino acid composition (AAC) is considered as the elementary feature in constructing the predictive model. Figure 3 displays the precision (Pre), sensitivity (Sn), specificity (Sp), accuracy (Acc), and Matthews Correlation Coefficient (MCC) resulting from the cross-validation evaluation done using various window lengths. When the window length varies from 9 to 21, it is observed that the predictive accuracy improves slightly from 0.749 to 0.808. As the window size increases, the predictive specificity improves while the sensitivity declines. Overall, the models trained using a window size of 15, 17, and 19 performs best. Given the consideration of both computational efficiency and predictive performance, 15-mer is chosen as the window length in the following analyses. According to the training feature of amino acid composition, the precision, sensitivity, specificity, accuracy, and MCC resulting from a model with a 15-mer window size are 0.696, 0.798, 0.814, 0.808, and 0.596, respectively.

### Evaluation of the effectiveness of studied features in identifying carboxylation sites

To explore which features can be used to effectively differentiate between carboxylation sites and non-carboxylation sites, the impact of three different features, such as amino acid sequence, ASA, and secondary structure, is evaluated using five-fold cross-validation. Amino acid sequences are encoded using a positional weighted matrix and the amino acid composition, denoted “AA_PWM” and “AAC”, respectively. The accessible surface area (ASA) and the secondary structure (SS) are encoded using the ASA values and a three-dimensional binary vector, respectively. Table 2 reveals that, among the models trained using a single feature, the model trained with a positional weighted matrix (AA_PWM) outperforms those trained with amino acid composition (AAC) alone, ASA alone or SS alone. The precision, sensitivity, specificity, accuracy, and MCC of the model with AA_PWM are 0.735, 0.817, 0.843, 0.834, and 0.646, respectively. The model trained with secondary structure alone performs worse.

**Table 2:** Cross-validation performance of the predictive models trained with various features.

Training features	Pre	Sn	Sp	Acc	MCC
Positional Weighted Matrix of flanking Amino Acids (AA_PWM)	0.735	0.817	0.843	0.834	0.646
Amino Acid Composition (AAC)	0.696	0.798	0.814	0.808	0.596
Accessible Surface Area (ASA)	0.672	0.768	0.800	0.789	0.553
Secondary structure (SS)	0.580	0.718	0.723	0.721	0.424
AA_PWM + AAC	0.738	0.814	0.846	0.835	0.647
AA_PWM + ASA	0.781	0.831	0.876	0.860	0.698
AA_PWM + SS	0.709	0.791	0.827	0.814	0.604
**AA_PWM + AAC + ASA**	**0.836**	**0.860**	**0.910**	**0.892**	**0.765**
AA_PWM + AAC + SS	0.711	0.801	0.827	0.818	0.613
AA_PWM + AAC + ASA + SS	0.812	0.860	0.894	0.882	0.745

Abbreviation: Pre, precision; Sn, sensitivity; Sp, specificity; Acc, accuracy; MCC, Matthews Correlation Coefficient.

Additionally, the predictive power of the model that is trained using a hybrid combination of AAC, AA_PWM, ASA, and SS is evaluated. With respect to the performance achieved using individual features, AA_PWM is crucial for training a model with other individual features. The model trained using the combination of AA_PWM and ASA substantially outperforms those trained with other combinations but slightly outperforms one that is trained with AA_PWM alone. Overall, the model trained with AA_PWM, AAC and ASA has the best accuracy. The predictive precision, sensitivity, specificity, accuracy, and MCC of the best model are 0.836, 0.860, 0.910, 0.892, and 0.765, respectively. However, some of the models that are trained with ASA or SS do not outperform that trained with AA_PWM alone. In conclusion, five-fold cross-validation indicates that the model that is trained using a combination of AA_PWM, AAC and ASA performs best, and is therefore adopted in further independent testing.

### Evaluation of γ-glutamyl carboxylation predictive model using independent test set

To test the effectiveness of the studied features yielding the highest accuracy in cross-validation, the independent set is utilized to test the model trained with a 15-mer window length and the features positional weight matrix (AA_PWM), amino acid composition (AAC), and accessible surface area (ASA). The independent test set comprises 60 carboxylation sites and 99 non-carboxylation sites in 14 proteins, none of which is included in the training data. Table 3 shows that the predictive sensitivity falls slightly during independent testing and specificity falls by around 10%. Overall, independent testing reveals that the model has an accuracy of 0.823, which approximates to that of cross-validation. The precision, sensitivity, specificity, and MCC in independent testing are 0.735, 0.833, 0.818, and 0.638, respectively. Accordingly, independent testing demonstrates that the positional weight matrix (AA_PWM), amino acid composition (AAC), and accessible surface area can distinguish between carboxylation and non-carboxylation sites when data are truly blind to the cross-validation process.

**Table 3:** Comparison of performances between cross-validation and independent testing.

	Five-fold cross validation	Independent testing
Number of positive data	302	60
Number of negative data	567	99
True Positive	260	50
False Positive	42	10
True Negative	516	81
False Negative	51	18
Precision	0.836	0.735
Sensitivity	0.860	0.833
Specificity	0.910	0.818
Accuracy	0.892	0.823
Matthews Correlation Coefficient	0.765	0.638

### Investigation of functional domains in carboxylated proteins

To study the preferred functional domains in carboxylated proteins, the annotations in InterPro [43] are used. InterPro is an integrated resource that was developed initially as a means of rationalizing the complementary uses of PROSITE [44], PRINTS [45], Pfam [46] and ProDom [47] databases, to provide protein "signatures" such as protein families, domains and functional sites. Based on the annotations in InterPro release 28.0, 134 carboxylated proteins had annotations for 32 InterPro functional types. Table 4 shows the InterPro annotations which occur in more than ten carboxylated proteins. The most featured annotation is the abundance of a “gamma-carboxyglutamic acid-rich (GLA) domain” (InterPro ID: IPR000294), which occurrs in 43 carboxylated proteins, 21 and 17 of which are involved in the “coagulation factor” domain (InterPro ID: IPR002383) and the “bone matrix” family (InterPro ID: IPR002384), respectively. Another annotation in 19 carboxylated proteins is associated with the functional domains “peptidase S1/S6, chymotrypsin/Hap” (InterPro ID: IPR001254) and “serine/cysteine peptidase, trypsin-like” (InterPro ID: IPR009003). Eighteen and 15 carboxylated proteins have peptidase-related annotations, “peptidase S1A, chymotrypsin” family and “peptidase S1A, coagulation factor VII/IX/X/C/Z” family, respectively. Additionally, 17 carboxylated proteins are associated with an EGF-like domain, including “EGF-like region, conserved site” (InterPro ID: IPR013032), “EGF-like, type 3” (InterPro ID: IPR000742), and “EGF-like” (InterPro ID: IPR006210). Interestingly, these proteins are also involved in the post-translational modification of the “EGF-type aspartate/asparagine hydroxylation site” (InterPro ID: IPR000152). In conclusion, the carboxylated proteins could be categorized into three functional groups, which are Gla domain, pep**t**idase, and EGF-like proteins.

**Table 4:** Statistics of InterPro functional annotations in 134 carboxylated proteins.

InterPro ID	Type	Description	Number of carboxylated proteins
IPR000294	Domain	Gamma-carboxyglutamic acid-rich (GLA) domain	43
IPR002383	Domain	Coagulation factor, Gla domain	21
IPR001254	Domain	Peptidase S1/S6, chymotrypsin/Hap	19
IPR009003	Domain	Serine/cysteine peptidase, trypsin-like	19
IPR001314	Family	Peptidase S1A, chymotrypsin	18
IPR013032	Conserved site	EGF-like region, conserved site	17
IPR000742	Domain	EGF-like, type 3	17
IPR000152	PTM	EGF-type aspartate/asparagine hydroxylation site	17
IPR006210	Domain	EGF-like	17
IPR002384	Family	Bone matrix, Gla protein	17
IPR018114	Active site	Peptidase S1/S6, chymotrypsin/Hap, active site	16
IPR006209	Domain	EGF	16
IPR018097	Conserved site	EGF-like calcium-binding, conserved site	15
IPR012224	Family	Peptidase S1A, coagulation factor VII/IX/X/C/Z	15
IPR001881	Domain	EGF-like calcium-binding	14

## Conclusions

Although the importance of carboxylation in the blood clotting cascade, bone growth and extraosseous calcification has been established, studies on carboxylation are still subject to technical limitations - especially when the concern is regarding substrate site specificity in γ-glutamyl carboxylation. Following the collection of experimentally verified carboxylation sites from UniProtKB and HPRD, 463 experimentally verified carboxylation sites have been identified in 134 carboxylated proteins. In this investigation, after the construction of a training data set and an independent test data set, the composition of the flanking amino acids among the training data is studied. The analysis of position-specific differences in amino acid composition reveals that the most prominent feature is the abundance of a uniform residue of glutamate around carboxylation sites. Another important characteristic is the slight increase in the abundance of arginine (positively charged amino acid) at positions -7, -5, -4, -1, and +3. This investigation indicates that the distant amino acids in the primary sequence, which may be immediately adjacent to the γ-carboxyglutamate (Gla) residue in a three-dimensional structure, differ notably between carboxylation sites and non-carboxylation sites.

Structural characteristics such as solvent-accessible surface area and secondary structure are also investigated. In the study of ASA curves, the region from -7 to -4 exhibits notable differences between carboxylation sites and non-carboxylation sites. The mean percentage of ASA values on the carboxylated glutamate residues is 37.8% which shows that it is greatly exposed to the solvent. The analysis of the secondary structure shows that the flanking regions of the carboxylated sites are likely to be present on the coil (loop) and helix structures. The five-fold cross-validation evaluation demonstrates that the SVM model trained using the combined features of positional weighted matrix, amino acid composition, and accessible surface area has the highest accuracy. Additionally, independent testing reveals that the proposed model can differentiate carboxylation sites from non-carboxylation sites when the data are truly blind to the cross-validation process.

Although the independent tests verify that the proposed method performs accurately and robustly, some issues warrant further investigation. First, the evolutionary conservation of carboxylation sites should be examined. The analysis of functional domains (See Supplementary Information) shows that carboxylated proteins can be categorized into three main functional groups: Gla domain, peptidase, and EGF-like proteins. However, carboxylated proteins with the same functional domain may be homologous. The carboxylation sites of homologous proteins should exhibit evolutionary conservation. Second, the structural affinities of carboxylation sites, particularly when flanking amino acids are not conserved, should be studied in greater detail [48,49]. The intrinsic disordered regions, B-factor, protein linker region, and other factors should also be explored at the vicinity of carboxylation sites in PDB entries. Finally, the biological function of carboxylated proteins should be investigated. Analysis of gene ontology [50], the occurrence of other PTMs, and the network context of protein-protein interaction [51] may offer a clue to the functions of carboxylated proteins.

## Competing interests

The authors declare that they have no competing interests.

## Authors’ contributions

TYL conceived and supervised the project. CTL, SAC and NAB were responsible for the design, data preprocessing, computational analyses, and drafted the manuscript with revisions provided by TYL. THC, MGS and KYH participated in the design of the study and performed the computational analysis. All authors read and approved the final manuscript.
